# Intraosseous basivertebral nerve ablation: A 5-year pooled analysis from three prospective clinical trials

**DOI:** 10.1016/j.inpm.2024.100529

**Published:** 2024-12-13

**Authors:** Jad G. Khalil, Eeric Truumees, Kevin Macadaeg, Daniel T.D. Nguyen, Gregory A. Moore, Dylan Lukes, Jeffrey Fischgrund

**Affiliations:** a Orthopaedic Surgery, Oakland University, William Beaumont School of Medicine, Department of Orthopaedic Surgery, Beaumont University Hospital, 3811 West 13 Mile Rd, Royal Oak, MI, 48073, USA; b Orthopaedic and Neurological Surgery, University of Texas, Dell Medical School, Ascension Spine & Scoliosis Center, Ascension Seton Medical Center, 1004 West 32nd Street #200, Austin, TX, 78705, USA; c Indiana Spine Group, 13225 N Meridian St, Carmel, IN, 46032, USA; d Indiana University School of Medicine, Department of Anesthesiology, IN, USA; e Comprehensive Specialty Care, Neuroradiology & Pain Solutions of Oklahoma, 1023 Waterwood Parkway, Edmond, OK, 73034, USA; f Pacific Sports and Spine, 217 Division Avenue, Eugene, OR, 97404, USA; g Statistics & Data Management, 730 Second Avenue South, Suite 500, Minneapolis, MN, 55402, USA; h Department of Orthopaedic Surgery, William Beaumont University Hospital, 3811 West 13 Mile Rd, Royal Oak, MI, USA

**Keywords:** Chronic low back pain, Basivertebral nerve, Intraosseous radiofrequency ablation, Modic changes, Vertebrogenic pain

## Abstract

**Background:**

Vertebrogenic pain is a documented source of anterior column chronic low back pain (CLBP) that stems from damaged vertebral endplates. Nociceptive signals are transmitted by the basivertebral nerve (BVN) and endplate damage is observed as Type 1 or Type 2 Modic changes (MC) on magnetic resonance imaging (MRI). The clinical impact and safety of intraosseous radiofrequency ablation of the BVN (BVNA) for the treatment of vertebrogenic pain has been demonstrated in three prospective clinical trials (two randomized and one single-arm study).

**Objective:**

Report aggregate long-term BVNA outcomes at five years from three studies.

**Methods:**

Pooled results at 5-years post-BVNA are reported for three clinical trials with similar inclusion/exclusion criteria and outcomes measurements: 1) a prospective, open label, single-arm follow-up of the treatment arm of a randomized controlled trial (RCT) comparing BVNA to sham ablation (SMART); 2) a prospective, open label, single-arm follow-up of the treatment arm of an RCT comparing BVNA to standard care (INTRACEPT); and 3) a prospective, open label, single-arm long-term follow-up study of BVNA-treated participants (CLBP Single-Arm). Paired datasets (baseline and 5-years) for mean changes in Oswestry disability index (ODI) and numeric pain scores (NPS) were analyzed using a two-sided paired *t*-test with a 0.05 level of significance. Secondary outcomes included responder rates, patient satisfaction, adverse events, and healthcare utilization.

**Results:**

Two hundred forty-nine (249) of 320 BVNA-treated participants (78 % participation rate) completed a five-year visit (mean of 5.6 years follow-up). At baseline, 71.9 % of these participants reported back pain for ≥5 years, 27.7 % were taking opioids, and 61.8 % had prior therapeutic lumbar spinal injections. Pain and functional improvements were significant at 5-years with a mean improvement in NPS of 4.32 ± 2.45 points (95 % CI 4.01, 4.63; p < 0.0001) from 6.79 ± 1.32 at baseline and a mean improvement in ODI of 28.0 ± 17.5 (95 % CI 25.8, 30.2; p < 0.0001) from 44.5 ± 11.0 at baseline. Nearly one-third (32.1 %) of patients reported being pain-free (NPS = 0) at five years, 72.7 % of patients indicated their condition improved and 68.7 % had resumed activity levels they had prior to onset of CLBP. In the sixty-nine participants taking opioids at baseline, 65.2 % were no longer taking them at 5-years, and spinal injections decreased by 58.1 %. The rate of lumbosacral treatment (therapeutic spinal injection, radiofrequency ablation, or surgery) for the same index pain source and vertebral level was 33/249 (13.2 %) at 5 years post BVNA; including a 6.0 % rate of lumbar fusion. There were no serious device or device-procedure related adverse events reported during the long-term follow-up.

**Conclusion:**

In this 5-year aggregate analysis, BVNA significantly improved pain and function scores compared to baseline. Similarly, there were significant reductions in opioid consumption and spinal injections post BVNA. Data demonstrate a strong safety profile with no serious device or device-related events and low healthcare utilization rate for the same index pain source through a mean of 5.6 years. Results demonstrate that intraosseous BVNA treatment for patients with vertebrogenic pain is safe, effective, and durable through five years.

## 1 Background

Historically, anterior column low back pain (LBP) was attributed to the intervertebral discs. Decades of basic science research have shifted the focus to the vertebral endplates as a notable source of anterior column pain [[1], [2], [3], [4], [5]].

The vertebral endplate is a bilayer of cartilage and bone separating the intervertebral discs and the adjacent vertebrae [6]. When damaged edema and bone marrow intensity changes (BMIC), or Modic Changes (MC), are visualized on magnetic resonance imaging (MRI) [7]. Pain signals from damaged vertebral endplates are transmitted via the BVN, a branch of the sinuvertebral nerve, which becomes thinly or non-myelinated after entering the vertebral body through the foramen at the posterior wall [3,4].

The diagnosis of vertebrogenic pain is made when a patient's clinical presentation is consistent with anterior column pain and MRI demonstrates edema and/or fatty infiltrative bone marrow changes (Type 1 and/or Type 2 MC) at the vertebral endplates [8]. Intraosseous radiofrequency ablation (RFA) of the BVN interrupts the transmission of pain signals from damaged vertebral endplates. The clinical impact and safety of basivertebral nerve ablation (BVNA) for the treatment of vertebrogenic LBP have been demonstrated in two randomized controlled trials (RCTs) [9,10], prospective single-arm cohort and long-term follow-up studies [[11], [12], [13], [14], [15], [16], [17], [18]], two aggregate analyses [19,20], and three independent meta-analyses [[21], [22], [23]].

In this analysis, we report the five-year post-BVNA pooled clinical outcomes data from three clinical trials; this allows a more comprehensive analysis of the durability and long-term clinical impact of BVNA in patients with vertebrogenic LBP.

## 2 Methods

### 2.1 Study design

The present study analyzed aggregate clinical outcomes and utilization data from three clinical trials sponsored by Relievant Medsystems, Inc. (Minneapolis, MN, USA). The three trials were (1) a prospective, double-blind, randomized sham-control trial (SMART) [9] with an optional prospective, open-label, single-arm follow-up study of the BVNA treatment arm with results published at 2- and 5-years [13,14], (2) a prospective, open-label RCT comparing BVNA to non-surgical standard care (INTRACEPT) [10,16] with an optional prospective, open-label, single-arm follow-up study of the BVNA treatment arm with results published at 2-years [17] and (3) a prospective, open-label, single-arm cohort study (BVNA Single Arm) [12,15] with an optional follow-up study. Pooled 3-year results for INTRACEPT and BVNA single-arm have been published previously [19] and all studies were included in a pooled healthcare utilization study previously [20]. Primary inclusion/exclusion criteria, study endpoints, and protocol requirements were similar for the three studies, allowing for data pooling.

Each study was registered on ClinicalTrials.gov: NCT01446419 (SMART), NCT03997825 (SMART 5+ Year Follow-up Study), NCT03246061 (INTRACEPT), NCT03266107 and NCT05207813 (BVNA Single Arm main study and Long-term Follow-up Study. The studies were compliant with Health Insurance Portability and Accountability Act (HIPAA), Good Clinical Practices, and the Declaration of Helsinki, and were conducted under Institutional Review Board approval and participant informed consent.

### 2.2 Study population

All participants enrolled in the three original studies had anterior column CLBP for a minimum of six months, with Type 1 and/or Type 2 MC at L3-S1. Baseline MRIs, pain body diagrams, and inclusion/exclusion were reviewed by up to three independent orthopedic spine medical reviewers to confirm Type 1 and/or Type 2 MC and the dominant pain source as vertebrogenic pain.

Participants in the three studies were identified from existing practice populations, physician referrals, and patient self-referrals and were enrolled between October 2011 and February 2019 at more than thirty study sites in the United States (US) and three in Europe. After voluntary informed consent, they were enrolled, randomized (SMART and INTRACEPT) and treated with BVNA. In SMART, patients were randomized 2:1 to BVNA versus sham ablation. For INTRACEPT, patients were randomized 1:1 to BVNA or non-surgical standard care. For the single-arm cohort study (BVNA Single Arm), all patients were treated with BVNA.

The primary inclusion and exclusion criteria for the three pooled studies are listed in Table 1. Enrollment criteria allowed for moderate spinal stenosis without associated neurogenic claudication symptoms, disc extrusions/protrusions ≤5 mm, and spondylolisthesis ≤2 mm. SMART excluded participants with prior lumbosacral surgery while the INTRACEPT and BVNA Single Arm studies allowed for previous non-fusion lumbar spine surgery (e.g., discectomies and laminectomies), if the surgery was >6 months prior to baseline enrollment and there were no ongoing radicular symptoms. The BVNA Single Arm study allowed the inclusion of patients with extended-release opioid use and BMI >40 (provided the procedure could be technically completed).

**Table 1:** Inclusion and exclusion criteria. The primary inclusion and exclusion criteria for the three pooled studies are outlined below with footnotes for any differences. Compared to the two RCTs, the BVNA Single Arm study allowed enrollment of patients taking extended-release opioids and BMI >40.

Inclusion Criteria	Exclusion Criteria
• Skeletally mature patients with chronic (≥6 months) isolated lumbar back pain, who had not responded to at least 6 months of non-operative management • Type 1 or Type 2 Modic changes at one or more vertebral body for levels L3-S1 • Minimum ODI of 30 points (100-point scale) • Minimum VAS of 4 cm (10 cm scale) | • Ability to provide informed consent, read and complete questionnaires	• MRI evidence of Modic changes at levels other than L3-S1 • Radicular pain (defined as nerve pain following a dermatomal distribution and that correlates with nerve compression in imaging) • Previous lumbar spine surgery (discectomy/laminectomy allowed if > 6 months prior to baseline and radicular pain resolved)^a^ • Symptomatic spinal stenosis (defined as the presence of neurogenic claudication and confirmed by imaging) • Metabolic bone disease, spine fragility fracture history, trauma/compression fracture, or spinal cancer • Spine infection, active systemic infection, bleeding diathesis • Radiographic evidence of other pain etiology: • Disc extrusion or protrusion >5 mm • Spondylolisthesis >2 mm at any level • Spondylolysis at any level • Facet arthrosis/effusion correlated with facet-mediated LBP • Beck Depression Inventory >24 or 3 or more Waddell's signs • Compensated injury or litigation • Currently taking extended-release opioids with addiction behaviors^b^ • BMI >40^b^ • Bedbound or neurological condition that prevents early mobility or any medical condition that impairs follow-up^a^ • Contraindication to MRI, allergies to components of the device, active implantable devices, pregnant or lactating

Abbreviations: MRI - magnetic resonance imaging; ODI - Oswestry Disability Index; VAS - Visual Analogue Scale (average low back pain in past 7 days); mm - millimeters; BMI - body mass index.

a Prior discectomy and laminectomy allowed in INTRACEPT and BVNA Single Arm only. SMART excluded all patients with previous lumbar spine surgery.

b Exclusion criteria for the SMART and INTRACEPT only.

### 2.3 Follow-up visits

The SMART and INTRACEPT main study protocols required up to 2-years of follow-up, with timepoints at 6-weeks and 3-, 6-, 12- and 24-months for SMART and at 6-weeks and 3-, 6-, 9-, 12- and 24-months for INTRACEPT. The BVNA Single Arm main study had one-year of follow-up with timepoints at 6-weeks and 3-, 6-, 9- and 12-months. Each study had an optional long-term follow-up study of the BVNA-treated participants. Main study participants were approached at their 24-month study visit (INTRACEPT) or by telephone at the 3-year visit timeframe (BVNA Single Arm) or five-year visit timeframe (SMART and INTRACEPT crossover participants). Long-term visits were performed at 3-, 4-, and 5-years (INTRACEPT and BVNA Single Arm) and 5-years (SMART 5+ Year Follow-up). Patients who were treated with BVNA from all three pooled studies had common follow-up timepoints at 3-, 6-months, and 5-years post BVNA.

The INTRACEPT trial included a pre-specified interim analysis conducted by an independent physician/statistician data management committee (DMC). The DMC recommended stopping enrollment early to offer cross to BVNA treatment for standard care arm participants based on superiority of all primary and secondary endpoints. Standard care arm participants were offered BVNA treatment at their next study visit after re-baseline measurements of the study endpoints. Sixty-one of the remaining 66 (92 %) standard care arm patients opted for BVNA. Crossover participants were followed for 3 and 6-months post BVNA. These participants were approached for voluntary consent for one long-term follow-up visit at 5 years post-BVNA. All crossover participants who had a 5-year visit are included in this pooled analysis with comparison to re-baseline values. Participants in the SMART control arm were allowed to cross to BVNA at the completion of the main study (with 73 % opting to receive BVNA). SMART crossover participants were followed for safety per the Food and Drug Administration and are not included in this pooled analysis. Table 2 outlines the follow-up visits for each study.

**Table 2:** Follow-up by individual pooled study.

Study	Baseline	2 Week	6 Week	3 Month	6 Month	9 Month	1 Year	2 Year	3 Year	4 Year	5 Year
SMART	O, U	O	M	O, U	O, U		O, U	O, U^a^			O, U
INTRACEPT (BVNA Arm)	O, U		M	O, U	O, U	O, U	O, U	O, U	O, U	O, U	O, U
INTRACEPT (SC Arm Cross to BVNA)^b^				O, U	O, U						O, U
BVNA Single Arm Study	O, U		M	O, U	O, U	O, U	O, U		O, U	O, U	O, U

Required follow-up visits for the pooled studies are outlined. All three studies had an optional long-term study where main study participants were re-consented. All three studies had follow-up at 3-months, 6-months, and 5 years post BVNA.

O = Follow-up visit with clinical outcomes (including Oswestry Disability Index and Visual Analog Scale/Numeric Pain Score and safety) collected.

U = Follow-up visit with utilization collected (including opioid medication utilization, spinal injections, lumbosacral radiofrequency ablation, and lumbosacral spine surgeries).

M = Follow-up visit with only magnetic resonance imaging (MRI) and adverse events collected.

Abbreviations: BVNA – basivertebral nerve ablation; SC – standard care.

a SMART utilization data collection included lumbosacral spine injections, lumbosacral radiofrequency ablation, and lumbosacral spine surgeries. No opioid data were collected at the 24-month visit for SMART.

b The INTRACEPT trial protocol defined a 3 and 6-month follow-up for standard care participants that elected to cross to BVNA. Participants were re-consented for a 5-year follow-up study.

### 2.4 Intervention

For each study included in this pooled analysis, BVNA was conducted within each vertebral body exhibiting Modic changes (L3 to S1) using the Intracept ® System (Relievant Medsystems, Minneapolis, MN USA). The full procedure has been described previously [[9], [10], [11]]. Any additional healthcare utilization for low back pain during study follow-up was based on shared decision-making between the participant and treating physician, and could have included physical therapy, exercise, chiropractic treatment, acupuncture, oral pain medications, lumbosacral spinal injections (LSI), lumbosacral radiofrequency ablation (LRFA), spinal cord stimulation (SCS), lumbosacral spine surgery (LSS), etc.

### 2.5 Data collection

Patient-reported Oswestry Disability Index (ODI) [24], Visual Analog Scale (VAS) [25], Short Form 36 Item Survey (SF-36) [26], and Beck Depression Index (BDI) [27] were collected at baseline using validated questionnaires for all three pooled studies. ODI was scored on a scale of 0 (no disability) to 100 (complete disability), with a minimal clinically important difference (MCID) of 15-points [28]. In the-long term follow-up studies, low back pain was assessed using a subject-reported 10-point numeric pain scale (NPS) that is based on the VAS pain rating questionnaire, where 0 represents no pain and 10 represents worst pain imaginable. NPS was collected as an average level of LBP experienced in the seven days prior to the study visit, with a MCID threshold of 2.0 cm/points, respectively [28]. Opioid use and historical LBP treatments were collected at baseline. Baseline data were independently monitored using participant medical records.

At five years, all three studies conducted a telephonic study visit. Data collection consisted of the five-year ODI, NPS, activity level, work status, patient satisfaction, opioid medication use (within the past 30 days), pain interventions, lumbosacral surgeries, and/or adverse effects. Medical records were requested for each patient or site-reported LBP pain intervention or surgery. Independent monitoring of medical records at the study site was conducted to ensure comprehensive reporting. Five-year data were collected via interview by an independent clinical research coordinator (SMART and the BVNA Single Arm) or by the study site clinical research coordinator (INTRACEPT). Study data were aggregated into a single validated clinical study database (Clindex version 5 software; Fortress Medical, Minneapolis, MN).

All post-BVNA LSIs (including epidural, intradiscal, trigger point, medial branch/facet joint, lateral branch/sacroiliac joint injections), pain interventions (therapeutic LRFA and SCS), and LSS were adjudicated by an independent Clinical Event Committee (CEC) comprised of two orthopedic spine surgeons. The CEC determined whether the involved levels and pain sources were the same for the additional treatments as the index vertebrogenic pain source and levels treated with BVNA. The treatments were conservatively deemed as treating the “same” pain if the source was unclear. For example, if therapeutic spinal injections were reported at the index level of BVNA, and no clear etiology was recorded in the medical records, it was determined to be treating the same treatment source and level.

### 2.6 Statistical analyses

Baseline characteristics are summarized using descriptive statistics. For categorical variables, the number (n) and percentages are reported. For continuous variables, the mean, standard deviation (SD), median, minimum, maximum, and confidence intervals are reported. To evaluate if statistical differences existed between studies, baseline demographic, clinical characteristics, and LBP treatments were analyzed and compared across individual studies. Pearson's Chi-Square test or the Exact Chi-Square test (when cell counts were less than five) was used for categorical variables, and the Omnibus F-test was used for continuous variables. All statistical testing to evaluate the poolability of the three full study populations for the five-year aggregate cohort used a two-sided 0.05 significance level.

Outcomes for pain (NPS) and function (ODI) were comparisons of baseline/re-baseline and five-year values. Clinical endpoints were analyzed as observed (no imputations for missing data), last observation carried forward (LOCF), and intent-to-treat (ITT), with missing data treated as failure (zero reduction from baseline). Pre-post analyses on paired data were conducted using McNemar's test for proportions and paired t-tests for means using a two-sided 0.05 significance level. Response rates at five years were analyzed using Pearson's Chi-Square test or the Exact Chi-Square test (when cell counts were less than five) with a two-sided 0.05 level of significance using published MCID thresholds of ODI ≥15-points improvement and NPS ≥2 points reduction [28]. Kaplan-Meier curves were generated to evaluate time to first LBP-related pain intervention/surgery following BVNA treatment. Results for patient satisfaction and healthcare utilization are summarized using descriptive statistics. Statistical analyses were performed with SAS version 9.4 software (SAS Institute, Cary, North Carolina) and R version 4.2.1 software (R Foundation for Statistical Computing, Vienna, Austria).

## 3 Results

### 3.1 Study participant disposition

**Fig. 1:**
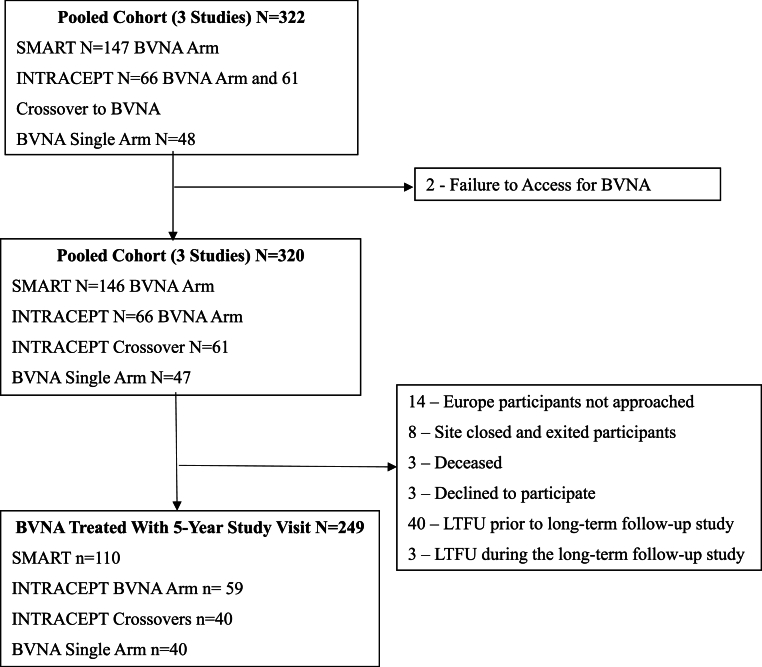
CONSORT diagram of participant disposition. The diagram reports the number of study participants enrolled in the prospective studies and in the 5-year pooled analysis. Reasons for participant study exit at each follow-up timepoint are reported. Per SMART main study protocol, participants were exited upon receiving lumbosacral intervention/surgery as a treatment failure. Abbreviations: BVNA - basivertebral nerve ablation; N – number; SC – standard care.

A combined total of 322 study participants with vertebrogenic pain (confirmed clinical inclusion criteria and the presence of Modic Type 1 and/or Type 2 changes on MRI) were enrolled at 30+ study sites and treated with BVNA by more than forty individual physicians who specialized in pain medicine, orthopedic surgery, anesthesiology, physical medicine and rehabilitation, sports medicine, neurosurgery, and/or neurointerventional radiology. Treatment was unsuccessful for two participants whose BVN could not be accessed during the procedure due to technical challenges with hard bone. Of the remaining 320 BVNA-treated study participants, 249 (78 %) had five-year follow-up visits and comprise the cohort for this pooled analysis. Fourteen participants in Europe (SMART study) were excluded as the long-term follow-up included only U.S. sites; eight (8) participants were excluded due to study site closure and subject exit at the completion of the main study; 40 participants were lost to follow-up prior to 5-year study visits; three (3) participants were deceased (unrelated to the study); three (3) declined to participate in the long-term follow-up, and three (3) were lost to follow-up during the 5-year data collection. Participants in the SMART sham control arm that crossed to BVNA at the completion of the main study were followed for safety only and are not included in the long-term pooled analysis. While individual studies had additional follow-up time points, the aggregate cohort had three common visits: 3-months, 6-months, and 5 years post-BVNA. Details on study participant disposition at each of these aggregate timepoints are reported in Fig. 1.

### 3.2 Pooled cohort baseline clinical characteristics

Baseline characteristics for participants are reported in Table 3 for both the pooled and individual study cohorts. The mean age was 48.5 years (range 26–71 years); 47.8 % were female, and 71.9 % had LBP for ≥5 years. One or more therapeutic LSI(s) were performed prior to baseline in 61.8 % of participants, 9.2 % were previously treated with LRFA of the facet joint(s), and 6.4 % had prior non-fusion lumbosacral surgery (discectomy/microdiscectomy or laminectomy/laminotomy) prior to baseline. Pooled study participants reported severe pain and disability scores at baseline with a mean VAS of 6.75 ± 1.34 and mean ODI of 44.5 ± 11.4. Anxiety and/or depression were reported by 32.1 % of study participants. Participants reported a low Quality of Life (QoL) at baseline with mean scores of 32.99 ± 6.85 and 52.39 ± 10.44 for SF-36 physical component score (PCS) and SF-36 mental component score (MCS), respectively.

**Table 3:** Baseline characteristics – pooled 5-year cohort.

Baseline Characteristics	SMART BVNA Arm 5-Year Cohort (N = 110)	INTRACEPT BVNA Arm 5-Year Cohort (N = 59)	INTRACEPT SC to BVNA 5-Year Cohort (N = 40)	BVNA Single Arm 5-Year Cohort (N = 40)	P-Value^a^	Pooled 5-Year Cohort (N = 249)
**Mean Age in Years Mean (Range))**	47.43 ± 10.89 (26.40, 69.90)	50.78 ± 9.61 (30.80, 68.50)	51.06 ± 10.77 (26.80, 71.00)	45.75 ± 8.53 (30.40, 66.60)	0.0249	48.54 ± 10.37 (26.40, 71.00)
**Female, n (%)**	43.6 % (48/110)	49.2 % (29/59)	47.5 % (19/40)	57.5 % (23/40)	0.5092	47.8 % (119/249)
**Duration LBP ≥ 5 years, n (%)**	69.1 % (76/110)	66.1 % (39/59)	87.5 % (35/40)	72.5 % (29/40)	0.1007	71.9 % (179/249)
**Pfirrmann grade, n (%)**						
**Pfirrmann grade <3**	1.8 % (2/110)	0.0 % (0/59)	2.5 % (1/40)	2.5 % (1/40)	0.7526	1.6 % (4/249)
**Pfirrmann grade 3**	16.4 % (18/110)	13.6 % (8/59)	15.0 % (6/40)	20.0 % (8/40)	0.8554	16.1 % (40/249)
**Pfirrmann grade 4**	41.8 % (46/110)	49.2 % (29/59)	20.0 % (8/40)	45.0 % (18/40)	0.0265	40.6 % (101/249)
**Pfirrmann grade 5**	40.0 % (44/110)	37.3 % (22/59)	62.5 % (25/40)	32.5 % (13/40)	0.0279	41.8 % (104/249)
**ODI,** **Mean ± SD (Range)**	43.33 ± 11.42 (30.00, 76.00)	44.75 ± 11.56 (30.00, 76.00)	44.63 ± 12.19 (26.00, 76.00)	47.05 ± 10.17 (30.00, 72.00)	0.3638	44.47 ± 11.40 (26.00, 76.00)
**VAS,** **Mean ± SD (Range)**	6.83 ± 1.46 (4.00, 10.00)	6.58 ± 1.23 (4.00, 9.00)	6.66 ± 1.40 (3.00, 9.00)	6.85 ± 1.07 (4.00, 9.20)	0.6304	6.75 ± 1.34 (3.00, 10.00)
**SF-36 (PCS),** **Mean ± SD (Range)**	33.48 ± 7.36 (14.83, 48.11)	32.57 ± 6.76 (18.43, 46.93)	33.04 ± 5.35 (24.65, 43.45)	32.21 ± 6.97 (18.62, 48.03)	0.7335	32.99 ± 6.85 (14.83, 48.11)
**SF-36 (MCS),** **Mean ± SD (Range)**	52.28 ± 10.54 (26.90, 69.06)	52.84 ± 9.52 (22.24, 69.80)	50.93 ± 10.80 (27.59, 66.25)	53.47 ± 11.29 (19.75, 68.03)	0.7252	52.39 ± 10.44 (19.75, 69.80)
**Beck Depression Index, Mean ± SD (Range)**	7.31 ± 5.70 (0.00, 23.00)	6.47 ± 5.36 (0.00, 20.00)	5.75 ± 4.29 (0.00, 16.00)	4.68 ± 3.79 (0.00, 13.00)	0.0377	6.44 ± 5.20 (0.00, 23.00)
**Anxiety/Depression, n (%)**	36.4 % (40/110)	30.5 % (18/59)	22.5 % (9/40)	32.5 % (13/40)	0.4438	32.1 % (80/249)

Baseline characteristics for participants are reported in Table 3 for both the pooled and individual study cohorts. While significant differences are noted in participant baseline age, Pfirrmann grades, and mean BDI score at 5-years, prior analyses report that these characteristics have limited predictive value of treatment outcome, and no adjustments were made for the pooled analysis.

Abbreviations: BDI – Beck Depression Index; BVNA - basivertebral nerve ablation; LBP - low back pain; MCS – mental component score; N/n - number; ODI – Oswestry Disability Index; PCS – physical component score; SF-36 - Short Form 36 Item Survey.

a P-values for categorical variables from Pearson's Chi-Square test. When expected cell counts were <5, then an Exact Chi-Square test was used. P-Values for continuous variables from Omnibus F-test for comparison of means.

Significant differences are noted in participant baseline age, Pfirrmann grades 4 and 5, and mean BDI scores between the pooled study populations. Prior predictive analyses showed age and Pfirrmann grade had no predictive value, while baseline BDI was a weak predictor of treatment failure [[29], [30], [31]]. Given the limited predictive value of the baseline characteristics that differed among the study populations, no adjustments were made for pooling of the populations (Table 3).

### 3.3 Baseline prior low back pain treatments – pooled 5-year cohort

LBP treatment history including LSI(s), LRFA(s), and lumbar surgeries anytime prior to baseline and opioid use at baseline for BVNA arm aggregate study participants (N = 249 with a 5-year follow-up) are reported by number and percent of participants for the pooled and individual studies. In all three studies, participants were required to have a minimum of six months of failed conservative treatment to be eligible for enrollment. There were significant differences between the three studies in the proportion of participants with prior intradiscal injections and lumbosacral surgeries. The SMART trial excluded potential participants with any prior lumbar spine surgeries while the INTRACEPT and BVNA Single Arm studies allowed for prior non-fusion lumbosacral procedures (e.g., discectomy, laminectomy) at the planned BVNA treatment level(s) with a required six-month post-surgery healing before study enrollment. As prior pain interventions and lumbosacral surgery suggest greater co-morbidity and potentially a higher risk of treatment failure, no adjustments were made for pooling of the data. Table 4 reports LBP treatments prior to baseline.

**Table 4:** Baseline low back treatments – pooled 5-year cohort.

Baseline Prior LBP Treatment	SMART BVNA Arm 5-Year Cohort (N = 110)	INTRACEPT BVNA Arm 5-Year Cohort (N = 59)	INTRACEPT SC to BVNA 5-Year Cohort (N = 40)	BVNA Single Arm 5-Year Cohort (N = 40)	P-Value^a^	Pooled 5-Year Cohort (N = 249)
**Opioid use at baseline, n (%)**	30.0 % (33/110)	35.6 % (21/59)	22.5 % (9/40)	15.0 % (6/40)	0.1173	27.7 % (69/249)
**Therapeutic LSIs prior to baseline, n (%)**^b^	60.0 % (66/110)	64.4 % (38/59)	72.5 % (29/40)	52.5 % (21/40)	0.2924	61.8 % (154/249)
**Epidural**	55.5 % (61/110)	52.5 % (31/59)	62.5 % (25/40)	45.0 % (18/40)	0.4559	54.2 % (135/249)
**Intradiscal**	0.0 % (0/110)	10.2 % (6/59)	2.5 % (1/40)	0.0 % (0/40)	0.0032	2.8 % (7/249)
**Facet Joint Injections**	15.5 % (17/110)	18.6 % (11/59)	20.0 % (8/40)	10.0 % (4/40)	0.5994	16.1 % (40/249)
**Nerve Root**	0.9 % (1/110)	1.7 % (1/59)	0.0 % (0/40)	7.5 % (3/40)	0.0798	2.0 % (5/249)
**SI Joint**	0.9 % (1/110)	1.7 % (1/59)	5.0 % (2/40)	0.0 % (0/40)	0.3199	1.6 % (4/249)
**Trigger point injections**	1.8 % (2/110)	3.4 % (2/59)	0.0 % (0/40)	0.0 % (0/40)	0.5135	1.6 % (4/249)
**Other**	0.9 % (1/110)	1.7 % (1/59)	5.0 % (2/40)	2.5 % (1/40)	0.5534	2.0 % (5/249)
**LRFA prior to baseline, n (%)**^2^	5.5 % (6/110)	16.9 % (10/59)	10.0 % (4/40)	7.5 % (3/40)	0.0958	9.2 % (23/249)
**Medial Branch**	5.5 % (6/110)	16.9 % (10/59)	10.0 % (4/40)	7.5 % (3/40)	0.0958	9.2 % (23/249)
**Lateral Branch**	0.0 % (0/110)	0.0 % (0/59)	0.0 % (0/40)	0.0 % (0/40)	N/A	0.0 % % (0/249)
**Basivertebral Nerve**	0.0 % (0/110)	0.0 % (0/59)	0.0 % (0/40)	0.0 % (0/40)	N/A	0.0 % % (0/249)
**Prior lumbosacral surgery, n (%)**^b^	0.0 % (0/110)	11.9 % (7/59)	7.5 % (3/40)	15.0 % (6/40)	0.0018	6.4 % (16/249)
**Discectomy/microdiscectomy**	0.0 % (0/110)	5.1 % (3/59)	7.5 % (3/40)	10.0 % (4/40)	0.0194	4.0 % (10/249)
**Laminectomy/Laminotomy**	0.0 % (0/110)	6.8 % (4/59)	2.5 % (1/40)	5.0 % (2/40)	0.0476	2.8 % (7/249)
**Total Disc Removal**	0.0 % (0/110)	0.0 % (0/59)	0.0 % (0/40)	0.0 % (0/40)	N/A	0.0 % % (0/249)
**Fusion**	0.0 % (0/110)	0.0 % (0/59)	0.0 % (0/40)	0.0 % (0/40)	N/A	0.0 % % (0/249)
**Neurostimulator**	0.0 % (0/110)	0.0 % (0/59)	0.0 % (0/40)	0.0 % (0/40)	N/A	0.0 % % (0/249)
**Other**	0.0 % (0/110)	1.7 % (1/59)	0.0 % (0/40)	0.0 % (0/40)	0.5582	0.4 % (1/249)

Table 4 reports low back treatments anytime preceding baseline and opioid use at baseline by individual group and pooled 5-year cohort. There were significant differences between the studies in the proportion of participants with prior intradiscal injections and lumbosacral surgeries. Prior LBP treatments suggest greater co-morbidity and potentially an increased risk of BVNA therapy failure, therefore, no adjustments were made for pooling of the data.

Abbreviations: BVNA - basivertebral nerve ablation; LBP – low back pain; LSI – lumbosacral spine injection; LRFA – lumbosacral radiofrequency ablation; N - number; SI – sacroiliac.

a P-values for categorical variables from Pearson's Chi-Square test. When expected cell counts were <5, then an Exact Chi-Square test was used.

b Categories are not mutually exclusive so rows may not sum to unique patient count provided.

### 3.4 Basivertebral nerve ablation (BVNA) treatment - pooled 5-year cohort

All vertebral levels exhibiting MC were required to be treated in the three pooled studies. A blinded, independent interventional radiologist confirmed treatment of all vertebral levels with Modic, as well as targeting success of treated levels. Table 5 reports the vertebral levels treated for each study and the aggregate cohort. Overall targeting success rates for the pooled 5-year cohort were 93.2 % (patients) and 96.8 % (vertebral bodies). All BVNA-treated participants (regardless of targeting success) with a 5-year visit are included in these results. Nearly all participants in this pooled cohort received BVNA treatment at L5 (98.4 %) while 70.7 % of participants had treatment at S1. L4 was treated in 46.2 % of pooled study participants, and 9.2 % were treated at L3. There were significant differences in vertebral levels treated between the three studies. While the SMART trial allowed for the treatment of 3 consecutive vertebral bodies from L3-S1, the other two studies allowed for the treatment of four consecutive vertebral bodies from L3-S1. In the SMART trial fewer L3 and L4 levels were treated and more S1 levels were treated than the other two studies. Prior analyses did not find the location of MC to be predictive of treatment response for BVNA [31]. Table 5 reports vertebral levels treated for the 5-year cohort.

**Table 5:** Vertebral levels treated - pooled 5-year cohort.

Vertebral Bodies Treated	SMART BVNA Arm 5-Year Cohort (N = 110)	INTRACEPT BVNA Arm 5-Year Cohort (N = 59)	INTRACEPT SC to BVNA 5-Year Cohort (N = 40)	BVNA Single Arm 5-Year Cohort (N = 40)	P-Value^a^	Pooled 5-Year Cohort (N = 249)
**L3, n (%)**	4.5 % (5/110)	10.2 % (6/59)	25.0 % (10/40)	5.0 % (2/40)	<0.0001	9.2 % (23/249)
**L4, n (%)**	40.0 % (44/110)	55.9 % (33/59)	52.5 % (21/40)	42.5 % (17/40)	<0.0001	46.2 % (115/249)
**L5, n (%)**	98.2 % (108/110)	98.3 % (58/59)	100.0 % (40/40)	97.5 % (39/40)	0.5040	98.4 % (245/249)
**S1, n (%)**	76.4 % (84/110)	61.0 % (36/59)	70.0 % (28/40)	70.0 % (28/40)	0.0002	70.7 % (176/249)

All vertebral levels exhibiting MC were confirmed as treated in the three pooled studies. Table 5 reports the levels treated for each individual study and the pooled cohort. Nearly all participants were treated at vertebral level L5 (98.4 %) and 70.7 % were treated at S1.

Abbreviations: BVNA - basivertebral nerve ablation; L – lumbar level; N – number; S - sacral.

a P-values for categorical variables from Pearson's Chi-Square test. When expected cell counts were <5, then an Exact Chi-Square test was used.

### 3.5 Pooled analysis 5-year pain (NPS) and function (ODI) results

In the pooled analysis of BVNA-treated patients with a 5-year visit, statistically significant improvements in pain (NPS) and function (ODI) were observed at a mean of 5.6 years (range 4.5–7.8 years) when compared to baseline. Mean changes in ODI and NPS were assessed for the following: as observed (no imputations for missing data), last observation carried forward (LOCF), and intent-to-treat (ITT) with missing data considered failure (improvement of zero from baseline values). In the as observed analysis, BVNA-treated participants reported a mean improvement in ODI of 28.0 ± 17.5 (p < 0.0001; 95 % CI 25.83, 30.19) from a baseline score of 44.5 ± 11.4 at five years post-BVNA. Mean improvement in NPS was 4.32 ± 2.45 (p < 0.0001; 95 % CI 4.01–4.63) from a baseline score of 6.8 ± 1.3 to 2.43 ± 2.30 at five years post-BVNA. Mean improvements in ODI and NPS were also significant in the LOCF and ITT analyses (see Table 6, Table 7).

**Table 6:** – Numeric Pain Score (NPS) Baseline to 5 Years – Pooled 5-Year Cohort Paired t-tests demonstrated significant improvements (p < 0.0001) from baseline to 5 years for NPS for all three analyses conducted: 1) as observed, 2) last observation carried forwa**r**d (LOCF), and 3) intention-to-treat (where missing data were deemed failure with a zero reduction from baseline).

	Baseline VAS Mean ± SD Range 95 % CI	5-Year NPS Mean ± SD Range 95 % CI	Change in NPS Baseline to 5-Years Mean ± SD Range 95 % CI	P-value^a^
**As Observed (N** = **249)**	6.75 ± 1.34 3, 10 6.58–6.92	2.43 ± 2.30 0, 8 2.14–2.72	4.32 ± −2.45 −2, 10 4.01–4.63	p < 0.0001
**LOCF**^b^**(N** = **320)**	6.79 ± 1.32 3, 10 6.65–6.94	2.79 ± 2.57 0, 10 2.51–3.07	4.01 ± 2.64 −3, 10 3.72–4.30	p < 0.0001
**ITT**^c^**(N** = **320)**	6.79 ± 1.32 3, 10 6.65–6.94	3.43 ± 2.83 0, 10 3.12–3.74	3.36 ± 2.81 −2, 10 3.05–3.67	p < 0.0001

Abbreviations: BVNA - basivertebral nerve ablation; CI - confidence interval; ITT – Intent to Treat; LOCF – last observation carried forward; N - number; NPS – numeric pain score; SD - standard deviation; VAS - visual analog scale.

a P-value calculated using two-sided paired *t*-test.

b One standard care participant that crossed to BVNA had a re-baseline but did not have a follow-up visit. The re-baseline value was carried forward for the LOCF analysis.

c ITT missing data indicated as a zero change from baseline.

**Table 7:** – Oswestry Disability Index (ODI) Baseline to 5 Years – Pooled 5-Year Cohort Paired t-tests demonstrated significant improvements (p < 0.0001) from baseline to 5 years in ODI for all three analyses conducted: 1) as observed, 2) last observation carried forward (LOCF), and 3) intent-to-treat (where missing data was deemed a failure with a zero reduction from baseline).

	Baseline ODI Mean ± SD Range 95 % CI	5-Year ODI Mean ± SD Range 95 % CI	Change in ODI Baseline to 5-Years Mean ± SD Range 95 % CI	P-value^a^
**As Observed (N** = **249)**	44.47 ± 11.40 26, 76 43.05–45.89	16.46 ± 15.07 0, 62 14.58–18.34	28.01 ± 17.46 −28, 76 25.83–30.19	p < 0.0001
**LOCF**^b^**(N** = **320)**	44.50 ± 11.01 26, 76 43.29–45.71	18.29 ± 15.93 0, 62 16.54–20.05	26.20 ± 18.15 −28, 76 24.21–28.20	p < 0.0001
**ITT**^c^**(N** = **320)**	44.50 ± 11.01 26, 76 43.29–45.71	22.70 ± 18.27 0, 66 20.69–24.71	21.80 ± 19.31 −28, 76 19.67–23.92	p < 0.0001

Abbreviations: BVNA - basivertebral nerve ablation; CI - confidence interval; ITT – Intent to Treat (missing data treated as zero change from baseline); LOCF – Last Observation Carried Forward; N - number; ODI – Oswestry Disability Index; SD - standard deviation.

a P-value calculated using two-sided paired *t*-test.

b One standard care participant that crossed to BVNA had a re-baseline but did not have a follow-up visit. The re-baseline value was carried forward for the LOCF analysis.

c ITT missing data indicated as a zero change from baseline.

### 3.6 Pain (NPS) response rates – pooled 5-year cohort

Response rates using published MCIDs definitions for pain and function [28] as well as clinically accepted definitions are outlined in Table 8, Table 9 for all three analyses conducted. Using the published MCID for pain (≥2-point improvement in NPS from baseline/re-baseline) [28], the proportion of responders at 83.1 % was significantly greater than the proportion of non-responders (p < 0.0001). See Table 8.

**Table 8:** – Numeric Pain Score (NPS) Response Rates – Pooled 5-Year Cohort Pearson's Chi-Squared tests demonstrated significant difference in response compared to non-response from baseline to 5 years using the published MCID for pain scores (≥2-point NPS reduction) and clinically relevant thresholds of NPS reductions of ≥30 % and/or ≥ 50 % for all three analyses conducted with the exception of the ITT analysis at the NPS ≥50 % reduction threshold where 50.9 % of the participants responded but statistical significance was not reached.

	≥2-point NPS reduction Yes % (n/N) P-Value^a^	≥30 % NPS reduction Yes % (n/N) P-Value^a^	≥50 % NPS reduction Yes % (n/N) P-Value^a^	100 % NPS reduction Yes % (n/N) P-Value^a^
**As Observed (N** = **249)**	83.1 % (207/249) p < 0.0001	81.5 % (203/249) p < 0.0001	65.5 % (163/249) p < 0.0001	32.1 % (80/249) p < 0.0001
**LOCF**^b^**(N** = **320)**	78.1 % (250/320) p < 0.0001	75.6 % (242/320) p < 0.0001	60.9 % (195/320) p = 0.0001	29.1 % (93/320) p < 0.0001
**ITT**^c^**(N** = **320)**	64.7 % (207/320) p < 0.0001	63.4 % (203/320) p < 0.0001	50.9 % (163/320) p = 0.7799	25.0 % (80/320) p < 0.0001

Abbreviations: BVNA - basivertebral nerve ablation; CI - confidence interval; ITT – Intent to Treat (missing data treated as zero change from baseline); LOCF – last observation carried forward; N - number; NPS – numeric pain score; SD - standard deviation.

a P-values for categorical variables from Pearson's Chi-Square test. When expected cell counts were <5, then an Exact Chi-Square test was used.

b One standard care participant that crossed to BVNA had a re-baseline but did not have a follow-up visit. The re-baseline value was carried forward for the LOCF analysis.

c ITT missing visit or data indicated as a zero change from baseline.

**Table 9:** – Oswestry Disability Index (ODI) Response Rates – Pooled 5-Year Cohort Pearson's Chi-Squared tests demonstrated significantly greater participant response than non-response from baseline to 5 years using published MCID [28] for ODI (MCID of ≥15-point improvement) and at a higher threshold of ≥20-point reduction for all three analyses conducted.

	≥15-point ODI reduction Yes % (n/N) P-Value^a^	≥20-point ODI reduction Yes % (n/N) P-Value^a^
**As Observed (N** = **249)**	78.3 % (195/249) p < 0.0001	73.5 % (183/249) p < 0.0001
**LOCF**^b^**(N** = **320)**	72.2 % (231/320) p < 0.0001	66.3 % (212/320) p < 0.0001
**ITT**^c^**(N** = **320)**	60.9 % (195/320) p = 0.0001	57.2 % (183/320) p = 0.0118

Abbreviations: Abbreviations: BVNA - basivertebral nerve ablation; ITT – Intent to Treat (missing data treated as zero change from baseline); LOCF – Last Observation Carried Forward; N/n - number; ODI – Oswestry Disability Index.

a P-values for categorical variables from Pearson's Chi-Square test. When expected cell counts were <5, then an Exact Chi-Square test was used.

b One standard care participant that crossed to BVNA had a re-baseline but did not have a follow-up visit. The re-baseline value was carried forward for the LOCF analysis.

c Missing data deemed no change from baseline in the ITT analysis.

**Fig. 2:**
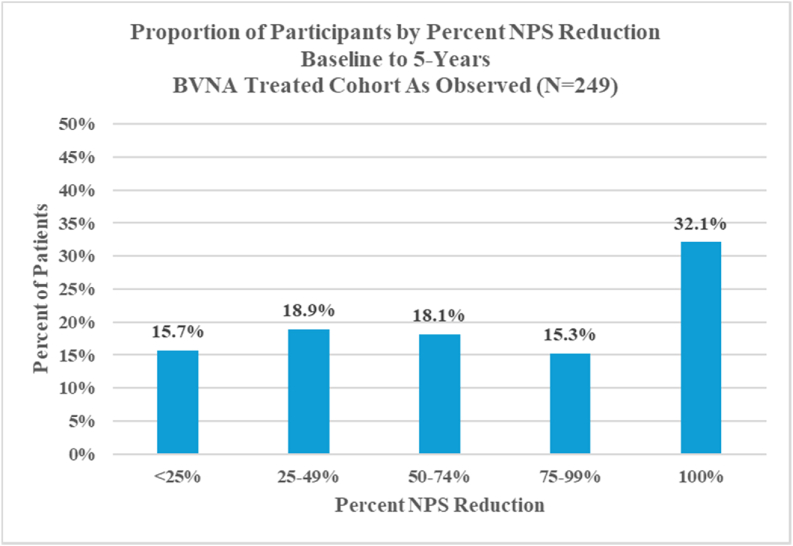
Pain response by quadrant – pooled 5-year cohort. Sixty-five percent (65.5 %) of participants reported a ≥50 % improvement in NPS at five years (p < 0.0001), while 47.4 % of participants reported a ≥75 % reduction, and 32.1 % of participants were pain-free (with a 100 % NPS reduction from baseline/re-baseline) at five years in the as observed analysis. Abbreviations: BVNA - basivertebral nerve ablation; NPS – numeric pain score.

The proportion of 5-year cohort participants reporting a ≥50 % improvement in NPS at five years was 65.5 % (p < 0.0001), while 47.4 % of participants reported a ≥75 % reduction, and 32.1 % of participants were pain-free (a 100 % NPS reduction from baseline/re-baseline) for the as observed analysis (Fig. 2).

### 3.7 Function (ODI) response rates – pooled 5-year cohort

Using the MCID threshold [28] for functional improvement, 78.3 % of the 5-year cohort participants reported a ≥15-point reduction in ODI (p < 0.0001) while 73.5 % reported a ≥20-point improvement (p < 0.0001). In an ITT analysis of functional improvement from baseline/re-baseline, where early dropouts are treated as non-responders, significant differences were demonstrated with 60.9 % of participants achieved ≥15 points of ODI improvement (p = 0.0001) and 57.2 % reported ≥20 points of improvement (p = 0.0118). See Table 9.

### 3.8 Composite response rates – pooled 5-year cohort

Composite endpoints based on published MCID (VAS ≥2 cm and ODI ≥15 points improvement from baseline/re-baseline), and other clinically relevant thresholds for pain and functional improvement (NPS ≥50 % reduction and ODI ≥15 points improvement from baseline/re-baseline), demonstrated significance response in paired analysis for the 5-year pooled cohort at 73.1 % (p < 0.0001) and 61.8 % (p = 0.0002) response rates respectively. LOCF analysis also demonstrated significance for both composite response definitions. While ITT analysis demonstrated significance for the MCID threshold of VAS ≥2 cm and ODI ≥15 points improvement (p = 0.0161), significance differences were not achieved for the composite threshold of ODI ≥15 points and NPS ODI ≥50 % improvement. See Table 10.

**Table 10:** – Composite ODI and NPS Results – Pooled 5-Year Cohort Significantly higher response rates were demonstrated in the “as observed” and LOCF analyses using both the published MCID composite response definition (VAS/NPS ≥2 cm/points and ODI ≥15 points improvement from baseline/re-baseline) and other clinically relevant definitions (VAS ≥50 % and ODI ≥15 points improvement from baseline/re-baseline). While ITT analysis did demonstrate significantly higher responders than non-responders at a composite threshold of VAS/NPS ≥2 cm/points and ODI ≥15 points improvement from baseline/re-baseline (p = 0.0161), significance was not achieved for the threshold of VAS ≥50 % and ODI ≥15 points improvement from baseline/re-baseline.

	≥15-point ODI reduction and ≥2-point NPS reduction Yes % (n/N) P-Value^a^	≥15-point ODI and ≥50 % NPS reduction Yes % (n/N) P-Value^a^
**As Observed (N** = **249)**	73.1 % (182/249) p < 0.0001	61.8 % (154/249) p = 0.0002
**LOCF**^b^**(N** = **320)**	66.6 % (213/320) p < 0.0001	56.9 % (182/320) p = 0.0161
**ITT**^c^**(N** = **320)**	56.9 % (182/320) p = 0.0161	48.1 % (154/320) p = 0.5387

Abbreviations: BVNA - basivertebral nerve ablation; ITT – Intent to Treat (missing data treated as zero change from baseline); LOCF – Last Observation Carried Forward; N/n - number; ODI – Oswestry Disability Index; NPS – numeric pain score.

a P-values for categorical variables from Pearson's Chi-Square test. When expected cell counts were <5, then an Exact Chi-Square test was used.

b One standard care participant that crossed to BVNA had a re-baseline but did not have a follow-up visit. The re-baseline value was carried forward for the LOCF analysis.

c ITT missing data indicated as a zero change from baseline.

### 3.9 Utilization results - pooled 5-year cohort

In the pooled 5-year cohort, statistically significant reductions in opioid use and therapeutic spinal injections were observed at a mean of 5.6 years (range 4.5–7.8 years) post-BVNA treatment. In the 5-year cohort, 69/249 participants (27.7 %) were taking opioids at baseline compared to 24/249 (9.6 %) taking opioids at 5 years, representing a reduction from baseline of 65.2 % (McNemar's test for proportions p < 0.0001; 95 % CI 63.6 %, 84.3 %).

Active use of LSI treatment was defined as one or more therapeutic lumbosacral injections within the 12-months preceding a study visit. In the pooled cohort, 33 % (83/249) of participants received 169 therapeutic LSIs within a period of 12 months preceding baseline. This proportion was reduced to 6.0 % (15/249) of the pooled participants receiving a total of 24 therapeutic LSIs in the 12 months preceding their 5-year study visit, a significant reduction in participants actively receiving LSIs post BVNA (p < 0.0001). Of the twenty-four therapeutic LSIs performed in the 12-months prior to the 5-year visit, only 3 (12.5 %) were adjudicated by the independent CEC to be for the same vertebrogenic pain source and/or vertebral treatment level.

An overall comparison of pre- and post-BVNA therapeutic LSI utilization was made using a period of five years for the 5-year pooled cohort. In this comparison, 55.8 % (139/249) of participants received 356 therapeutic LSIs in the five years preceding baseline compared to 22.9 % (57/249) of the participants receiving a total of 149 therapeutic LSIs in the mean of 5.6 years post-BVNA; showing 33 % fewer participants treated with LSIs (p < 0.0001) and a 58.1 % reduction in LSIs post BVNA. Of the 149 total therapeutic LSIs performed in the mean of 5.6 years following BVNA, only 34 (22.8 %) were adjudicated by the independent CEC to be for the same vertebrogenic pain source and/or vertebral treatment level.

Twenty-four (9.6 %) of the 5-year pooled cohort received 51 LRFAs (44-lumbar facet joint, 3-SI joint, and 4-additional BVNA) during the follow-up study. Of these, 8/51 (15.7 %) of the LRFAs (7-facet and 1-basivertebral nerve utilized in 3 patients) performed were conservatively adjudicated as treatment for the same vertebrogenic pain source and treatment level as the original BVNA. Three of the additional BVNAs performed were at a different treatment level than the original vertebral levels with MC documented at baseline. The remaining 43/51 (84.3 %) therapeutic LRFAs were adjudicated by the CEC as treatment for separate posterior element pain sources (based on response to the additional treatment).

Twenty-five of the five-year cohort participants (10.0 %) had thirty additional LSSs during the mean follow-up of 5.6 years post-BVNA. Of the 5-year pooled cohort participants, 3/249 (1.2 %) had four discectomies, 5/249 (2.0 %) had a laminectomy, 2/249 (0.8 %) had disc replacement, 1/249 (0.4 %) had SCS implanted, and 18/249 (7.2 %) had a lumbosacral spinal fusion. The independent CEC adjudicated 18 of the 30 additional lumbosacral surgeries as for the same pain source and/or treatment level as the original BVNA treatment (2 - total disc replacements, 1- neurostimulator implant, and 15 – lumbosacral spinal fusions). Of the 18 patients with a lumbosacral spinal fusion, 15 were adjudicated as the same etiology and/or pain source; of note, eight of the fifteen fusions (53 %) were performed at one study site, while the remaining were performed across seven other study sites.

Additional lumbosacral procedures that were adjudicated by the CEC to be for a different pain source or treatment level than the original vertebrogenic pain source or BVNA treatment level(s) included four – discectomies (2 – at different levels than the BVNA, 2 – for new disc herniations deemed unrelated to the BVNA treatment), five laminectomies (2 - new disc herniations, 2 - radiculopathy due to stenosis, and 1 – neurogenic claudication), and three lumbosacral fusions (1- motor vehicle accident trauma, 1 - disc collapse, and 1-for treatment of spinal stenosis at a different level than BVNA).

Overall, 78/249 (31.3 %) of participants had additional lumbosacral treatments (lumbosacral injections, RFA, or spine surgery) in the mean of 5.6 years of follow-up. Twenty-nine participants (11.6 %) had one or more procedures that were adjudicated as treatment for the same vertebrogenic pain source and level(s); with 15 of these participants having lumbar fusion.

### 3.10 Utilization survival curves - pooled 5-year cohort

**Fig. 3:**
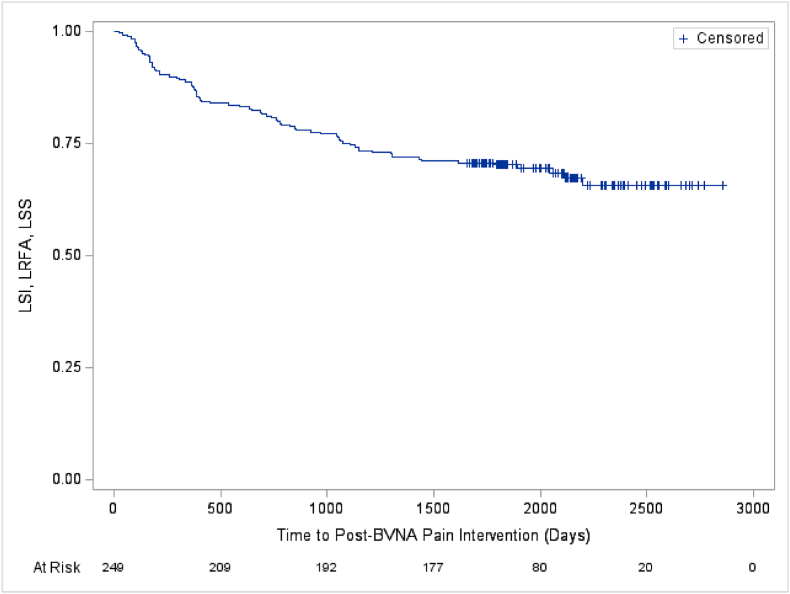
All lumbosacral treatments post basivertebral nerve ablation (BVNA). Fig. 3 - Depicts the days post-BVNA to first additional lumbosacral therapeutic pain intervention [spinal injection (LSI), lumbosacral radiofrequency ablation (LRFA), and/or lumbosacral spine surgery (LSS)] in the 5-year cohort of N = 249. LSIs accounted for most of the additional lumbosacral interventions. Approximately 69 % of participants were lumbosacral treatment-free through a mean of 5.6 years post-BVNA. ^a^ Note: The mean survival time and its standard error are underestimated as the longest observation was censored and the estimation was restricted to the longest event time. Abbreviations: BVNA – basivertebral nerve ablation; LSI – lumbosacral spine injection; LRFA – lumbosacral radiofrequency ablation; LSS – lumbosacral spine surgery.

**Fig. 4:**
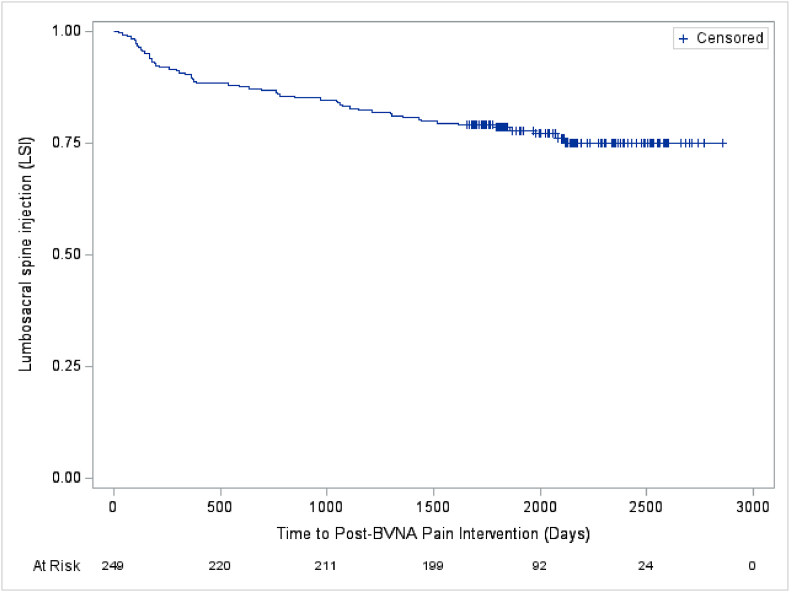
Lumbosacral spinal injections post basivertebral nerve ablation (BVNA). Fig. 4 - Depicts the days post-BVNA to first lumbosacral spinal injection (LSI) in the pooled analysis of N = 249 participants with a 5-year follow-up. Approximately 77 % of participants were LRFA-free through a mean of 5.6 years post-BVNA. ^a^ Note: The mean survival time and its standard error are underestimated as the longest observation was censored and the estimation was restricted to the longest event time. Abbreviations: BVNA – basivertebral nerve ablation; LSI – lumbosacral spinal injection.

**Fig. 5:**
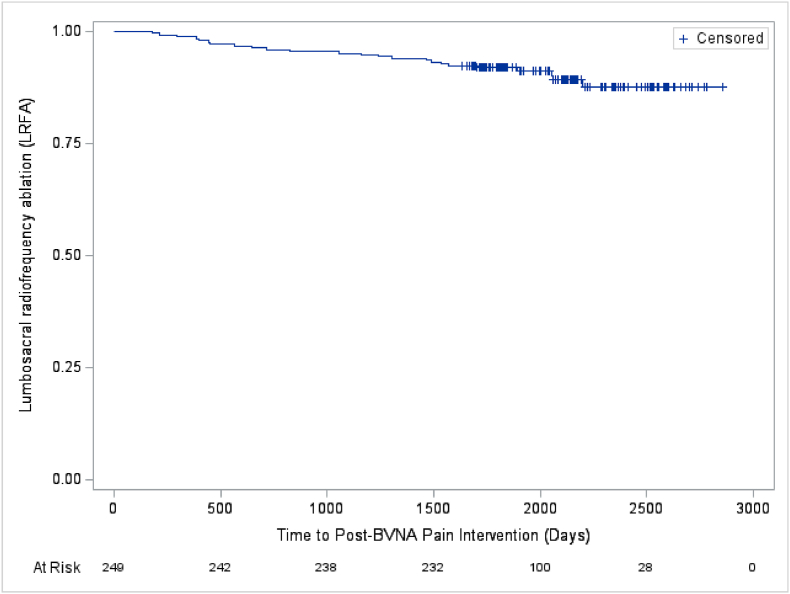
–Lumbosacral radiofrequency ablation (LRFA) post basivertebral nerve ablation (BVNA). Fig. 5 - Depicts the days post-BVNA to first lumbosacral radiofrequency ablation (LRFA) in the pooled analysis of N = 249 participants with a 5-year follow-up. Approximately 90 % of participants were LRFA-free through a mean of 5.6 years post-BVNA. ^a^ Note: The mean survival time and its standard error are underestimated as the longest observation was censored and the estimation was restricted to the longest event time. Abbreviations: BVNA – basivertebral nerve ablation; LRFA – lumbosacral radiofrequency ablation.

**Fig. 6:**
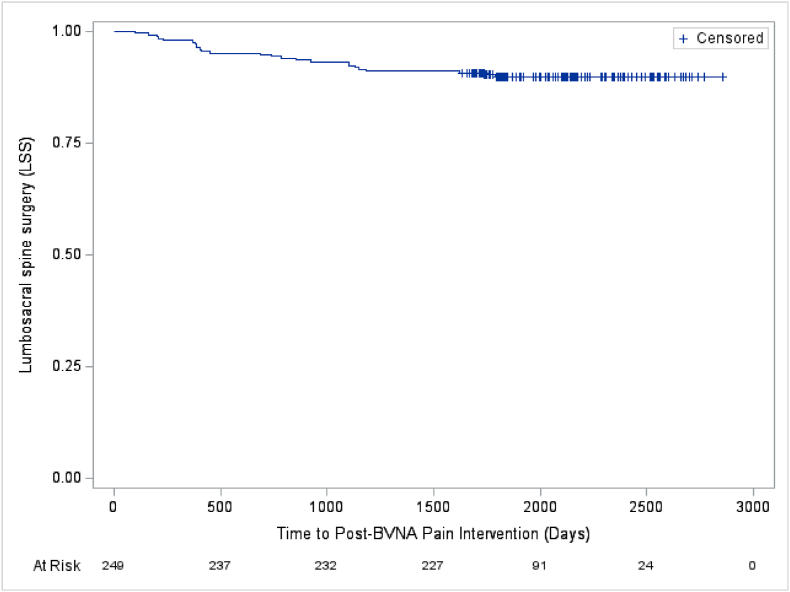
–Lumbosacral spine surgery post basivertebral nerve ablation (BVNA). Fig. 6 - Depicts the days post-BVNA to first lumbosacral spinal surgery in the pooled analysis cohort of N = 249 participants with a 5-year follow-up. Approximately 90 % percent of participants were lumbosacral spine surgery-free through a mean of 5.6 years post-BVNA. ^a^ Note: The mean survival time and its standard error are underestimated as the longest observation was censored and the estimation was restricted to the longest event time. Abbreviations: BVNA – basivertebral nerve ablation; LRFA – lumbosacral spine surgery.

Kaplan-Meier analyses were used to determine the average time to first LBP-related treatment after BVNA in the 249 cohort participants with a five-year follow-up. The Kaplan-Meier survival curves depict the probability of not having a treatment event (therapeutic LSI, LRFA, or LSS) over time after BVNA. The x-axis represents days since BVNA; and the y-axis represents the proportion of participants who have not had a lumbosacral pain intervention/lumbosacral surgery at each timepoint. The number at risk at the bottom of each figure is a count of the number of participants with follow-up data at the specific timepoint and thus who are still at risk of having a lumbosacral treatment post-BVNA. Participants are removed from this “risk pool” upon experiencing a lumbosacral treatment event (therapeutic LSI, LRFA, or LSS) or if they left the study before experiencing an event (censored). See Fig. 3, Fig. 4, Fig. 5, Fig. 6.

### 3.11 BVNA as the sole treatment - pooled 5-year cohort

To further isolate the BVNA treatment effect, an analysis comparing two groups (BVNA as the sole treatment and BVNA plus additional lumbosacral spine treatment) was performed. Five-year pooled participants that had additional lumbosacral spine treatments did not perform better than those with BVNA alone. No significant differences in ODI and NPS improvements were noted between participants that received BVNA treatment alone compared to participants that received BVNA treatment plus one or more additional lumbosacral treatment (LSI, LRFA, LSS) during the mean of 5.6 years post-BVNA period. While the participants that had additional lumbosacral treatments for LBP reported a lower response for pain when using a threshold of ≥50 % reduction in NPS from baseline to 5-years post BVNA, the rate was not significantly different than those participants that were treated with BVNA alone (p = 0.1459). See Table 11.

**Table 11:** – BVNA Alone versus BVNA Plus Additional Pain Treatment Five-year pooled participants that had additional lumbosacral spine pain treatments did not perform better overall than those with BVNA alone. No significant differences in ODI and NPS improvements or response rates were noted between pooled participants that received BVNA alone compared participants that received BVNA and one or more additional lumbosacral treatment (LSI, LRFA, LSS) during the mean of 5.6 years post-BVNA period.

	BVNA + Treatment^a^ (N = 78)	BVNA Alone (N = 171)	P-Value^b^	BVNA Treated Pooled 5-Year Cohort (N = 249) P-Value
**Change in ODI Baseline to 5-Years** Mean ± SD Range 95 % CI	25.54 ± 17.11 −8, 64 21.68–29.40	29.14 ± 17.55 −28, 76 26.49–31.79	0.1314	28.01 ± 17.46 −28, 76 25.83–30.19 p < 0.0001
**Change in NPS Baseline to 5-Years** Mean ± SD Range 95 % CI	4.17 ± 2.63 −2, 9 3.58–4.76	4.39 ± 2.37 −1. 10 4.03–4.75	0.5136	4.32 ± 2.45 −2, 10 4.01–4.63 p < 0.0001
**ODI Response Rate (reduction of ≥15-points from baseline)** % (n/N) of responders	74.4 % (58/78)	80.1 % (137/171)	0.3065	78.3 % (195/249) p < 0.0001
**NPS Response Rate (reduction of ≥ 50 % in NPS from baseline)** % (n/N) of responders	59.0 % (46/78)	68.4 % (117/171)	0.1459	65.5 % (163/249) p < 0.0001
**Composite Response Rate (reduction of ≥15-points ODI and ≥50 % in NPS from baseline)** % (n/N) of responders	56.4 % (44/78)	64.3 % (110/171)	0.2329	61.8 % (154/249) 0.0002

Abbreviations: BVNA – basivertebral nerve ablation; LSI – lumbosacral spine injection; LRFA – lumbosacral radiofrequency ablation; LSS – lumbosacral spine surgery; N/n – number; NPS – numeric pain scores; ODI – Oswestry disability index.

^c^Paired *t*-test.

a Lumbosacral treatments include LSI, LRFA, LSS.

b Two-sample *t*-test comparison of means for ODI and NPS reductions and Pearson's Chi-Square test for comparison for a given responder outcome.

### 3.12 Participant satisfaction - pooled 5-year cohort

Nearly 73 % of the 5-year pooled cohort participants who received BVNA reported improvement after their BVNA procedure (7.2 % slightly improved, 18.1 % much improved, and 47.7 % vastly improved). At five years post-BVNA, 21.7 % indicted “no change” and 5.6 % indicated their LBP had worsened. Seventy-four percent of participants indicated they were satisfied with the results of the BVNA procedure and 80 % indicated they would have the BVNA procedure again for the same condition. At their 5-year visit, 171 (68.7 %) indicated they were able to resume the level of activity they enjoyed prior to the onset of their LBP.

### 3.13 Safety - pooled 5-year cohort

Early in the post-op follow-up periods of the main studies, there were 28 non-serious device-procedure related events reported in the 473 patients treated (5.9 % of patients); the most common events were leg pain – all were mild to moderate and transient in nature, resolving within 48.5 days (median). A single serious adverse event (a vertebral compression fracture) was reported in the main SMART study and resolved without sequelae [9]. No serious device or device-procedure related adverse events were reported in this pooled long-term follow-up study.

## 4 Discussion

We report statistically significant and clinically meaningful improvements in paired analyses from baseline through five years post-BVNA in this pooled analysis. All three analyses (as observed, LOCF, and ITT) demonstrated statistically significant improvements in pain (NPS) as well as function (ODI) at a mean of 5.6 years (range 4.5–7.8 years) post BVNA demonstrating the durability of this therapy. In this pooled study population of severely impacted participants (ODI of 44.5 ± 11.0 and VAS of 6.8 ± 1.3 at baseline), improvements of twice the published MCID for both pain and function were demonstrated [(ODI improvements of 28.0 ± 17.5 (p < 0.0001; 95 % CI 25.83, 30.19) and NPS reductions of 4.32 ± 2.45 (p < 0.0001; 95 % CI 4.01–4.63)] at more than 5 years after treatment with BVNA. Similar significant improvements in pain and function were reported for the LOCF and ITT, where all participants without a 5-year follow-up are indicated as a zero change from baseline.

Response rates for functional improvement were high with 78.3 % of the 5-year pooled cohort participants reporting a ≥15-point reduction in ODI and a 60.9 % ODI response rate for that same threshold in the ITT analysis (p = 0.0001). Observed pain response rates using a high threshold of reduction of ≥50 % in NPS were significant at 65.5 % (p < 0.0001) while a response rate of 50.9 % was noted even in ITT analysis where main study participants that did not participate in the optional 5-year follow-up were conservatively deemed a non-responder. These pooled results are compelling for the treatment of vertebrogenic pain with intraosseous BVNA, particularly when the harshest metric of ITT demonstrates significant improvements in ODI and NPS at five years post BVNA. Similar results have been reported in an independently conducted prospective single arm cohort study [18] and independent meta-analyses at 12-months [22] that we continue to see at five-years post BVNA.

Another indicator of the clinical impact of BVNA involves the demonstrated reduction in post ablation adjunct treatments (injections, surgery, etc.). All three study protocols allowed for treatment per physician discretion. Kaplan Meier survival analysis showed 69 % percent of pooled participants were without lumbosacral spine treatment (LSI, LRFA, LSS) through a mean 5.6 years post-BVNA. We observed a significant decrease in opioid utilization (65.2 % of patients taking opioids at baseline were taking them at 5-years (p < 0.0001). We also observed a significant decrease in participants receiving LSIs (59.0 % decrease in the 5 years pre-versus post-BVNA), with 13/249 (5.2 % of subjects) with spinal injections adjudicated by an independent CEC as treatment for the same index vertebrogenic pain and BVNA treatment level. The rate of lumbosacral fusions adjudicated as the same pain source and level as BVNA is low at 6.0 % of subjects, particularly given this population may been considered by some to be candidates for fusion prior to availability of BVNA. Six percent is less than half the published fusion rate of 14 % in a population of patients with isolated degenerative disc disease, axial-dominant CLBP symptoms, and no evidence of instability [32]. Rates of BVNA treatment “failure” (additional LSI, LRFA, LSS) may be overestimated in this report as the CEC adopted a conservative approach where participants that had additional treatment without a response were adjudicated as BVNA treatment failure.

In this pooled analysis, participants that had additional lumbosacral pain treatments (LSI, LRFA, LSS) post BVNA did not perform better than participants with BVNA alone, with no significant differences noted for ODI and NPS improvements or response rates during the 5-year post-BVNA period. Two thirds of the participants had prior lumbosacral treatments at baseline (61.8 % with prior therapeutic LSI, 9.2 % with prior LRFA, and 6.4 % with prior discectomy/microdiscectomy) with unresolved axial LBP. These data support that intraosseous BVNA is an appropriate first treatment consideration for patients with a clinical assessment consistent with anterior column pain and Type 1 or Type 2 Modic changes (vertebrogenic pain); even in multifactorial pain situations.

It is important to differentiate vertebrogenic pain from other potential sources of CLBP. This is done by clinical assessment of pain, exacerbating movements indicative of anterior spine pain, and previous LBP treatment history, in addition to imaging confirmation of Type 1 and/or Type 2 Modic changes, and/or features consistent with Modic such as inflammation, edema, vertebral endplate changes, disruption and fissuring of the endplate, etc. Vertebrogenic pain is often described as midline low back pain, typically without radiation, that is exacerbated by forward flexion and sitting [30]. The presence of Modic changes, when used as an objective imaging biomarker of vertebrogenic pain, was the only predictor of response to intraosseous BVNA [29,31]. These pooled results support the use of Modic changes in combination with clinical characteristics that are consistent with vertebrogenic pain for intraosseous BVNA treatment decision-making.

We found it interesting that three participants were later treated with intraosseous BVNA at a different level than the index BVNA location(s). In each individual study, an independent interventional radiologist confirmed that every level that had Modic changes at baseline was treated by reviewing the 6-week post-ablation MRI scan; this suggests that new Modic changes occurred during the 5-year follow-up. As these results demonstrate the durability of BVNA treatment effect through five years, it may be prudent to consider additional endplate damage and evaluate surrounding levels for new Modic changes in patients who have recurrent anterior column pain after BVNA. Only one participant in this pooled analysis had a repeat BVNA of the index level and remained non-responsive, suggesting additional BVNA treatments of the same level may not be beneficial unless targeting or access were not achieved.

Access designed for safe, smooth, consistent, and accurate targeting of the BVN is crucial for achieving similar clinical outcomes to those reported here. These pooled study results are based on targeting at 30–50 % across the vertebral body and midline using a transpedicular approach. Transpedicular access achieved targeted location in 93 % of participants. Clinical outcomes in this pooled analysis are the result of BVNA treatment at every lumbosacral level with Modic changes. This pooled population included S1 treatment for 70.7 % of participants. Accessing S1 may be challenging without the proper tools designed to both curve around the iliac crest and to achieve a target at 30–50 % across the S1 vertebral body. Targeting for BVNA is closer to the posterior wall than other procedures such as vertebroplasty where a more anterior target (≥50 % across the vertebral body) is desired. The S1 was demonstrated to be safely accessed using a transpedicular approach in the more than 320 participants patients treated in this pooled analysis (with 225 of these including treatment at S1). We recommend transpedicular access based on the safety profile and targeting success rates for these pooled studies.

The pooled safety profile is favorable with no serious device or device-related adverse events reported in the long-term follow-up study. The most common non-serious event reported in the main pooled studies was leg pain early in the post op period. All leg pain events were treated with oral medication and were transient in nature. CEC review of the post-BVNA MRI found procedural trajectories that were too medial in nearly all patients complaining of post-procedure leg pain. While many of the study investigators were experienced in vertebroplasty, they had not performed BVNA prior to their first study procedure. Targeting is more posterior for BVNA than vertebroplasty and may account for the transient leg pain. Safety events were evaluated by specialty of the treating physician and number of cases performed with no patterns noted. Likewise, no differences in pain and functional outcomes for consecutive participants were noted in the studies suggesting a minimal learning curve for this procedure.

The strengths of this pooled analysis include a high participation rate of 78 % at a mean of 5.6 years post BVNA and the homogenous cohorts, with individual studies having similar inclusion/exclusion criteria, clinical endpoints, and study visit timeframes. While significant differences in age, BDI, and Pfirrmann grades were noted at baseline, previously published analyses have demonstrated these variables to have no or limited predictive value for treatment success/failure [[29], [30], [31]]. These pooled results across more than thirty academic and community study sites, with 40+ treating physicians specialized in pain medicine, orthopedic surgery, anesthesiology, physical medicine and rehabilitation, sports medicine, neurosurgery, and/or and neurointerventional radiology, along with similar findings in independent studies support the generalizability and reproducibility of these results.

Limitations of this pooled analysis are the open-label design, industry sponsorship (as it standard for new therapies), and a lack of long-term comparator with the high crossover rate of the standard care/sham arms in the two RCTs. Prior meta-analyses inclusive of these pooled studies as well as previous independent studies showed no evidence of serious publication bias per Egger's test [22]. More than 70 % of the participants in this pooled analysis reported CLBP for ≥5 years at baseline with over two-thirds having prior lumbosacral interventions/surgery. This amount of prior intervention in the five years preceding baseline for pooled participants (who continued to report severe pain and functional disability at baseline), suggest that continued non-BVNA treatment would not have demonstrated additional improvement at 5-years from baseline.

These five-year results demonstrate that a single treatment with intraosseous BVNA at each vertebral level with Modic changes in patients with vertebrogenic pain leads to successful patient outcomes, not requiring repeated treatment. We continue to observe a significant decrease in healthcare utilization post-BVNA. This includes a significant decrease in opioid usage and spinal injections. Similarly, there was a low likelihood of subsequent spinal fusion for the same pain source (6.0 %) in the pooled BVNA population. Since adjuvant interventional treatment offers little to no improvement, we recommend consideration of BVNA as first-line interventional treatment for patients with CLBP with anterior column pain and vertebral endplate changes. Unfortunately, non-responders to the therapy continue to exhibit little to no response to other modalities as well. This continues to be an area of potential further study.

## 5 Conclusions

This pooled analysis demonstrates significant and clinically meaningful improvements in pain and function that are durable through more than 5 years post BVNA in participants with vertebrogenic pain. Safety analysis for the three pooled clinical trials demonstrates a favorable safety profile with no serious device or device-related events in the follow-up studies. Similarly, we observe significant reduction in opioid consumption and post-BVNA spinal injections. Results from this pooled analysis support that treatment with intraosseous BVNA, for patients with vertebrogenic pain, is safe, effective, and durable through five years.

## Funding

This work was supported by Relievant Medsystems (Edina, MN), now Boston Scientific Corporation. All authors of this manuscript had full access to the data for each trial and determined pooled results independent of Relievant Medsystems.

## Conflict of interest statement

Jad G. Khalil, M.D. has received research funding from Relievant Medsystems (paid directly to the research institution Beaumont University Hospital) and serves as a consultant to Relievant Medsystems (now Boston Scientific). Eric Truumees, M.D. has received research funding from Relievant Medsystems, Orthofix, Stryker, and Medtronic (paid directly to the research institution, Ascension Seton Medical Center). Daniel T.D. Nguyen M.D. has received research funding from Relievant Medsystems and SI-Bone (paid directly to the research institution, Penn State University). He has served as a consultant for SI-Bone and Stratus Medical. Kevin Macadaeg, M.D. has received research funding from Relievant Medsystems (paid directly to the research institution, Indiana Spine Group) and serves as a consultant to Relievant Medsystems (now Boston Scientific). Gregory A. Moore, M.D. has received research funding from Relievant Medsystems (paid directly to the research institution Pacific Sports and Spine) and serves as a consultant to Relievant Medsystems (now Boston Scientific). Dylan Lukes, PhD, received research funding by Relievant Medsystems (paid directly to his organization Bright Research). Jeffrey Fischgrund, MD, serves as a consultant to Relievant Medsystems (now Boston Scientific) and served as the Primary Investigator and a Data Safety Monitoring Board member for the SMART randomized controlled trial (RCT) and Clinical Evaluation Committee (CEC) member for the INTRACEPT RCT; he did not enroll participants in the any of the three pooled studies.
